# Degradation Behavior of 3D-Printed Residue of Astragalus Particle/Poly(Lactic Acid) Biocomposites under Soil Conditions

**DOI:** 10.3390/polym15061477

**Published:** 2023-03-16

**Authors:** Wangwang Yu, Jianan Shi, Rui Qiu, Wen Lei

**Affiliations:** 1 School of Mechanical Engineering, Nanjing Vocational University of Industry Technology, Nanjing 210023, China; yuww@niit.edu.cn; 2 College of Science, Nanjing Forestry University, Nanjing 210037, China

**Keywords:** poly(lactic acid), Astragalus residue powder, biocomposite, fused deposition modeling, soil burial, degradation

## Abstract

Astragalus is widely cultivated in China, and the residue of Astragalus particles (ARP) can be used as reinforcements in fused filament-fabricated (FFF) natural fiber/Poly(lactic acid)(PLA) biocomposites. To clarify the degradation behavior of such biocomposites, 3D-printed 11 wt% ARP/PLA samples were buried in soil, and the effects of soil burial duration on the physical appearance, weight, flexural properties, morphology, thermal stability, melting, and crystallization properties were investigated. At the same time, 3D-printed PLA was chosen as a reference. The results showed that, with prolonged soil burial, the transparency of PLA decreased (but not obviously), while the surface photographs of ARP/PLA became gray with some black spots and crevices; especially after 60 days, the color of the samples became extremely heterogeneous. After soil burial, the weight, flexural strength, and flexural modulus of the printed samples all reduced, and greater losses happened to ARP/PLA pieces than pure PLA. With an increase in soil burial time, the glass transition, cold crystallization, and melting temperatures, as well as the thermal stability of PLA and ARP/PLA samples, all increased gradually. Additionally, soil burial had a greater effect on the thermal properties of ARP/PLA. The results showed that the degradation behavior of ARP/PLA was more significantly affected by soil burial than the behavior of PLA. Additionally, ARP/PLA more easily degraded in soil than PLA.

## 1. Introduction

Three-dimensional (3D) printing, also known as additive manufacturing, makes it possible to accelerate product prototyping and fabricate 3D parts with complex structures [1,2,3]. As a 3D printing technology, fused filament fabrication (FFF) 3D printing has attracted extensive attention from scientists and engineers in recent years because of its short design-to-manufacturing cycle, low material losses, technical simplicity, relatively low price, and potential applicability [4,5,6].

For FFF 3D printing, many research efforts are currently being directed towards developing new varieties of fully biodegradable feedstock materials that combine natural fibers with biodegradable polymers. For example, Charles et al. [7] produced flax/PLA filaments by coating continuous flax fiber with PLA in a 190 °C heated die and discussed the influence of humidity on the mechanical and failure properties of 3D-printed composites made by these filaments. Bright et al. [8] mixed powdered raw and alkali-treated pineapple leaf fibers (PALF) with PLA and extruded the mixture as filament for 3D printing. Agaliotis et al. [9] prepared henequen flour/PLA composite filaments by reinforcing PLA with 1–5 wt% henequen flour particles, and then printed samples with a 0° deposition angle. These FFF 3D-printed biocomposites have many advantages such as low cost, low density, acceptable specific mechanical properties, enhanced biodegradability, and recyclability.

Poly(lactic acid) (PLA) is a type of aliphatic polyester produced from 100% renewable resources. This material is biocompatible, compostable, reproducible, and can be degraded using H_2_O and CO_2_. PLA is also more environmentally stable than petroleum-based polymers such as polystyrene, polyvinyl chloride, and acrylonitrile butadiene styrene (ABS). In addition, PLA melts at a lower temperature than ABS and can adhere to the platform well. The low thermal expansion coefficient of this material also makes it resistant to warping. Meanwhile, there is no pungent smell when printing with PLA. For the above reasons, PLA has become the preferred raw material for FFF 3D printing [10,11]. However, PLA has some limitations. Most notably, the material’s high price (about 4500 USD/t in China compared to about 1750 USD/t for general plastics such as polyethylene and polypropylene) has become a major barrier to the commercialization of PLA-based products and also problematizes the wider application of PLA FFF technology.

Natural fibers are cheap, quickly renewable, and available worldwide. Additionally, such fibers have some unique properties such as low density, nonabrasive processing, high specific strength, and inherent biodegradability. Consequently, these fibers are often incorporated into biodegradable polymer matrixes via extrusion and injection processes to produce fully biobased composites with lower production costs by replacing a portion of the expensive PLA material without destroying the biodegradation performance of the matrix polymer [12,13,14]. The same is true for FFF 3D printing. For instance, Hao Liu [15] extracted cellulose fiber from abandoned crop sugarcane bagasse (SCB) and then FFF 3D-printed SCB/PLA biocomposites. After investigation, the authors found that the fully oriented sample printed using the “parallel” method had better tensile strength. Nasır Narlıoğlu [16] modified scotch pine wood flour (WF) using butyric anhydride and explored the effect of modification on the mechanical properties and thermal stability of FFF 3D-printed biocomposites. The authors found that both the tensile strength values and thermal stability improved, while the flexural strength and modulus decreased. David Stoof et al. [17] explored the feasibility and factors involved in using FFF technology to produce harakeke (Phormium tenax)/PLA composite components. The tensile test results of the printed samples demonstrated that the Young’s modulus and tensile strength of the composites were greater than those of plain PLA samples by 42.3% and 5.4%, respectively, indicating that harakeke is a useful fiber in terms of its mechanical properties.

As one of the most important Chinese traditional medicinal crops, Astragalus has been widely cultivated and used as a medicine for more than 2000 years [18]. Astragalus polysaccharides extracted from Astragalus were reported to have many biological activities, such as improving immune functions; facilitating myocardial preservation; limiting myocardial infarction size; inhibiting cardiac fibrosis; lowering blood sugar; and exerting anti-tumor, anti-inflammatory, increasing insulin sensitivity, and antioxidant activities [18,19,20]. Nevertheless, after extracting the drug components through cooking, the residue of Astragalus particles (ARP) is often disposed, which will not only pollute the environment but also create resource waste. To solve this problem, we once sought to apply ARP to FFF 3D printing and explored, in detail, the effects of printing parameters on the properties of the FFF 3D-printed ARP/PLA samples [21]. The properties of the ARP/PLA samples were also compared with those of the FFF 3D-printed wood flour/PLA and rice straw powder/PLA samples [22]. Our previous work demonstrated that ARP is an ideal candidate reinforcement for the FFF 3D printing of natural fiber/PLA biocomposites. Using ARP, the composite specimen was not only printed smoothly but also had correct comprehensive properties, such as moderate mechanical properties and thermal stability [21,22]. As a biocomposite, ARP’s degradability must be seriously considered. However, to the best of our knowledge, the degradation behavior of FFF 3D-printed ARP/PLA biocomposites has not been investigated or reported in the open literature. To fill this research gap and promote the further application of FFF 3D-printed ARP/PLA biocomposites, this paper focuses on the soil biodegradation of printed ARP/PLA samples and investigates the effects of soil burial on the physico-mechanical properties of the printed samples. The degradation behavior of the FFF 3D-printed PLA was also investigated for comparison.

## 2. Experimental Section

### 2.1. Materials and Reagents

**Figure 1:**
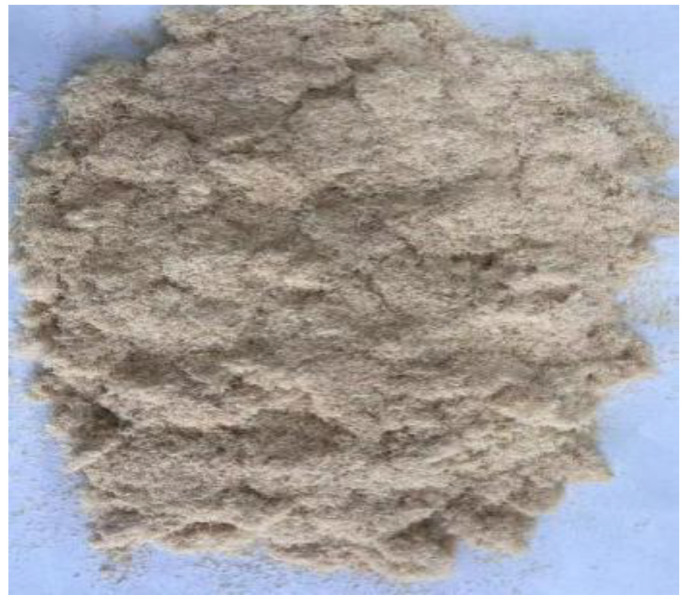
A real photo of ARP.

PLA was obtained from American Nature Works Co., b3052D, Minnetonka, MN, USA; ARP was passed through 120-mesh sieve made in our lab. The visual appearance of ARP is shown in Figure 1.

### 2.2. Preparation of FFF Filaments

For FFF 3D printing ARP/PLA pieces, both the cost and quality of the filament should be taken into consideration. The cost of the filament could be reduced gradually by using more ARP because this material is waste material after drug components are extracted, making it very cheap. However, we found it difficult for the ARP melt to flow and pass through the die during extrusion. Additionally, the quality of the filament was poor when the content of ARP in the composite totaled more than 11% wt. Filaments containing 11% wt ARP were thus prepared in this research.

ARP and PLA granules were dried at 80 °C for 8 h prior to extrusion to remove water. Two formulations were prepared: pure PLA and PLA with 11%-wt ARP, labeled as PLA and ARP/PLA, respectively. The blend of ARP and PLA was compounded with a twin screw extruder (SHJ-20, Nanjing Giant Machinery Co., Ltd., Nanjing, China) at 20 rpm and 130~160 °C. The extruded composites were then granulated to make pellets. For comparison, pure PLA granules were first extruded and pelletized. Then, the corresponding FFF filaments of PLA and ARP/PLA with a diameter of about 1.75 ± 0.05 mm were prepared using a twin-screw extruder (KS-HXY, Kunshan Huanxinyang Electrical Equipment Co., Ltd., Suzhou, China). The extrusion temperatures were set to 170 and 190 °C from hopper to die. The screw speed was set to 20 r/min.

### 2.3. FFF 3D Printing of Pieces

**Figure 2:**
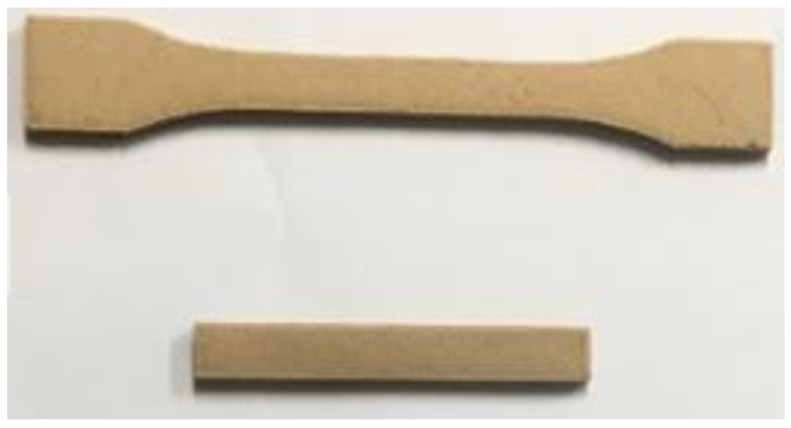
The printed ARP/PLA samples.

The prepared filaments were used for FFF 3D printing in a desktop-level 3D printer (MOSHU S10, Hangzhou Shining 3D Technology Co., Ltd., Hangzhou, China). The sample model file (STL file) for the test used computer-aided designed and was then sliced and transformed into G-code. The diameter of the 3D printer nozzle was 0.4 mm. The print temperature and speed were set to 220 °C and 50 mm/s, respectively, and the layer height was set to 0.1 mm. The 3D-printed pieces are shown in Figure 2.

### 2.4. Soil Burial

**Figure 3:**
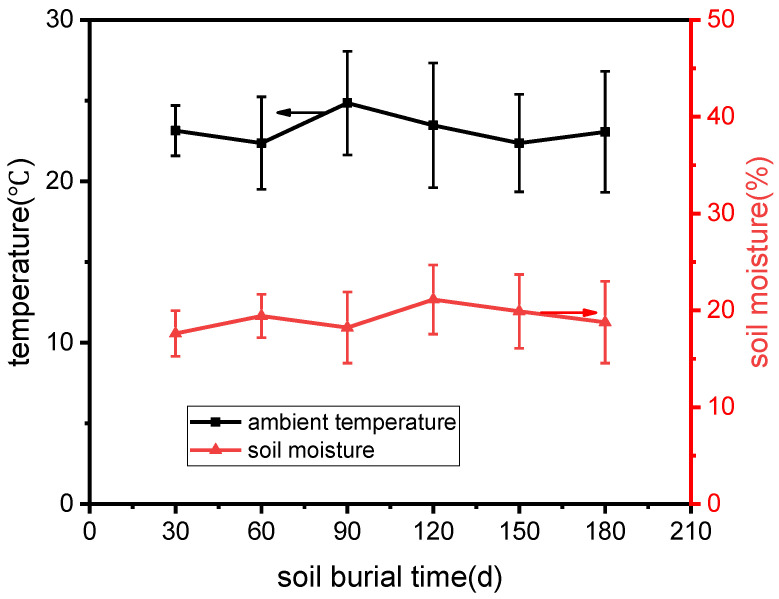
Average indoor temperature and soil moisture during soil degradation.

Natural soil was wetted and collected in a 30 × 20 × 20 cm^3^ container. Then, the samples were buried at 12 cm below the surface of the soil for up to 180 days. During soil burial, the moisture of the soil was measured and recorded every 5 days. Once the moisture was found to be lower than 15%, some water was added to the soil to keep the moisture between 15% and 25%. The average indoor temperature and soil moisture during the burial test are illustrated in Figure 3. At the desired times (30, 60, 90, and 180 days after burial), the buried specimens were dug from the soil, rinsed in water, and dried in a hot-air oven to a constant weight prior to taking photos; then, the samples were weighed and tested for their flexural properties and characterized.

### 2.5. Testing and Characterization

#### 2.5.1. Weight Loss

Weight measurement was performed using an analytical balance before and after soil burial at every regular time interval. The weight loss (WL) of each sample was calculated using the below formula:

(1) $$WL(\%)=\frac{w_{0}-w_{t}}{w_{0}}\times 100\%$$

where *w*_0_ is the initial dry weight before soil burial, and *w_t_* is the final dry weight after soil burial at exposure time *t*. The average of at least five measurements was calculated to obtain the reported results.

#### 2.5.2. Mechanical Testing

Flexural specimens were conditioned at room temperature (23 °C and 45% relative humidity) and measured using a three-point bending test according to the ASTM 790 standard testing method. A universal machine (E44.304, MTS Industrial Systems (China) Co., Ltd., Shenzhen, China) with a 20 kN load cell was used for testing. The crosshead speed for flexural testing was 5 mm/min.

#### 2.5.3. Morphological Observation (SEM)

Fractured samples from flexural testing were first sputter-coated with gold to avoid electrical charging during examination and then observed using a Hitachi SU 8010 field-emission scanning electron microscope (Hitachi Corporation, Tokyo, Japan) at an accelerated voltage of 3 kV.

#### 2.5.4. Thermal Stability

Evaluation of the thermal stability of the printed samples was carried out via thermogravimetric analysis (TG 209F1, NETZSCH-Gerätebau GmbH, Selb, Germany) on samples from 20 to 550 °C at a 20 K/min heating rate. TGA analysis was performed on approximately 5~12 mg of printed PLA and ARP/PLA under a nitrogen atmosphere.

#### 2.5.5. Melting and Crystallization Behavior

The glass transition (T_g_), melting (T_m_), and cold-crystallization (T_cc_) temperatures were determined using differential scanning calorimetry (DSC214, NETZSCH-Gerätebau GmbH, Selb, Germany) under a nitrogen flow of 20 mL/min. Samples for DSC analysis were cut from FFF 3D-printed PLA and ARP/PLA with a weight between 5 and 10 mg. The samples were heated from ambient temperature to 220 °C in a sealed aluminum pan at a ramp rate of 10 °C/min and held in an isothermal state for 5 min. Then, the samples were cooled to room temperature at a rate of 10 °C/min and subsequently reheated to 220 °C at a rate of 10 °C. Enthalpies of melting (∆*H_m_*) and cold crystallization (∆*H_cc_*) were evaluated using the NETZSCH analysis software by integrating the areas of the melting and cold crystallization peaks. The T_g_, T_m_, and T_cc_ values were taken from the second heating curves. The crystallization percentage of each piece was obtained via the following equation:

(2) $$x_{c}=\frac{\left|\Delta H_{m}-\Delta H_{cc}\right|}{\omega \Delta H^{*}}\times 100\%$$

where *ω* is the mass fraction of PLA in the piece, Δ*H_m_* is the melting enthalpy, Δ*H_cc_* is the cold crystallization enthalpy, and ΔH* = 93.7 J/g is the melting enthalpy of 100% crystalline PLA [23,24].

## 3. Results and Discussion

### 3.1. Physical Appearance

**Figure 4:**
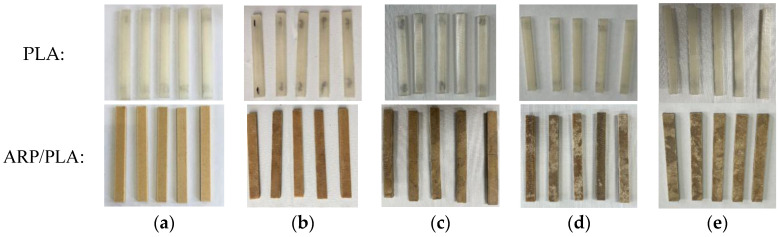
Photographs of PLA and ARP/PLA samples after different days of soil burial: (**a**) 0; (**b**) 30; (**c**) 60; (**d**) 90; (**e**) 180.

Figure 4 presents the typical physical appearance of both PLA and ARP/PLA specimens exposed to the soil environment for various intervals. Before soil burial, the surfaces of all the samples were smooth and homogeneously colored. The surfaces then changed to varying degrees during the degradation period.

As shown in Figure 4, the pure PLA did not present significant macroscopic alternations on the surface during the early degradation period. After 60 days, the surface of the pure PLA became slightly darkened (but not obviously), indicating the low degradation rate of PLA in the soil. This result was consistent with the slow biodegradation of PLA under natural soil [25,26]. However, the surface photographs of ARP/PLA became gray with some black spots and crevices throughout the soil burial degradation process. Especially after 60 days, the color of the samples became very heterogeneous, indicating an increased degradation rate with the introduction of ARP and a prolonged soil burial time. Compared with the pure PLA, after 60 days, the ARP/PLA samples degraded sharply with obvious physical surface changes, demonstrating more serious degradation of the ARP/PLA biocomposite samples in a soil environment.

### 3.2. Weight Loss

**Figure 5:**
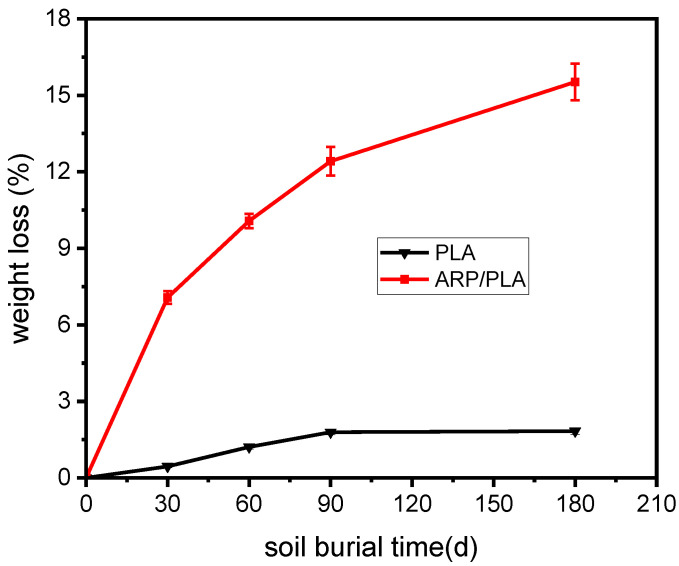
Weight loss curves of different specimens during degradation.

The weight loss percentage in the PLA and ARP/PLA samples after burying them for 30, 60, 90, and 180 days in soil is depicted in Figure 5, which shows that the fraction of weight loss in both the plain PLA and biocomposite samples presented in this work steadily increased with increased days of soil burial. We also found that the weight loss of PLA was very minor during the whole experiment; this result may be due to the hydrophobic properties of PLA, as well as the narrowly distributed microorganisms, which can degrade PLA in a soil environment. Figure 5 also shows that the printed ARP/PLA specimens always exhibited much greater weight loss than the PLA specimens with the same soil burial duration, meaning that ARP/PLA was more biodegradable than pure PLA. A greater difference in the weight loss fraction between the two samples was produced after a longer soil burial. After being buried in soil for 180 days, only 1.83% weight loss was observed in plain PLA, while 15.52% weight loss was observed for the ARP/PLA biocomposite samples. From the perspective of weight loss, the incorporation of ARP could accelerate the degradation of PLA in soil burial, which agrees with the results of the physical observations. The greater weight loss in ARP/PLA corresponds to an increased degradation rate due to the weak bonding between ARP and PLA, the easier degradation of ARP (a natural fiber) compared to PLA, and the synergetic effect due to the higher water adsorption by ARP [26].

### 3.3. Prediction of Flexural Properties

**Figure 6:**
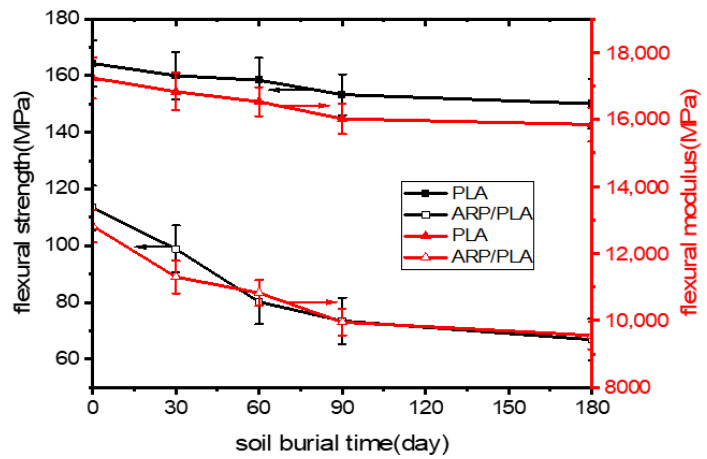
Bending properties of different specimens during degradation: (1) bending strength; (2) bending modulus.

The results of the flexural tests on pure PLA and ARP/PLA composites during the soil burial degradation test are presented in Figure 6. Both the flexural strength and flexural modulus of ARP/PLA showed far lower values compared to those of PLA. Meanwhile, the results depicted an obvious decreasing trend in the flexural strength and modulus of ARP/PLA composites with prolonged soil burial days. However, a much lower reduction in the flexural strength and modulus was observed for plain PLA. The loss in flexural strength for pure PLA and ARP/PLA after 180 days of soil burial was 8.64% and 41.01%, respectively, while that in the flexural modulus was 8.06% and 25.49%, respectively. As noted previously, we observed weak bonding between ARP and PLA in the composite samples. Additionally, ARP is a high cellulosic fiber that absorbs moisture much more readily during soil burial, leading to a weakening of fiber bonds [26]. As a result, the flexural strength and modulus of ARP/PLA reduced obviously from those of pure PLA.

### 3.4. Cross-Sectional Morphologies

**Figure 7:**
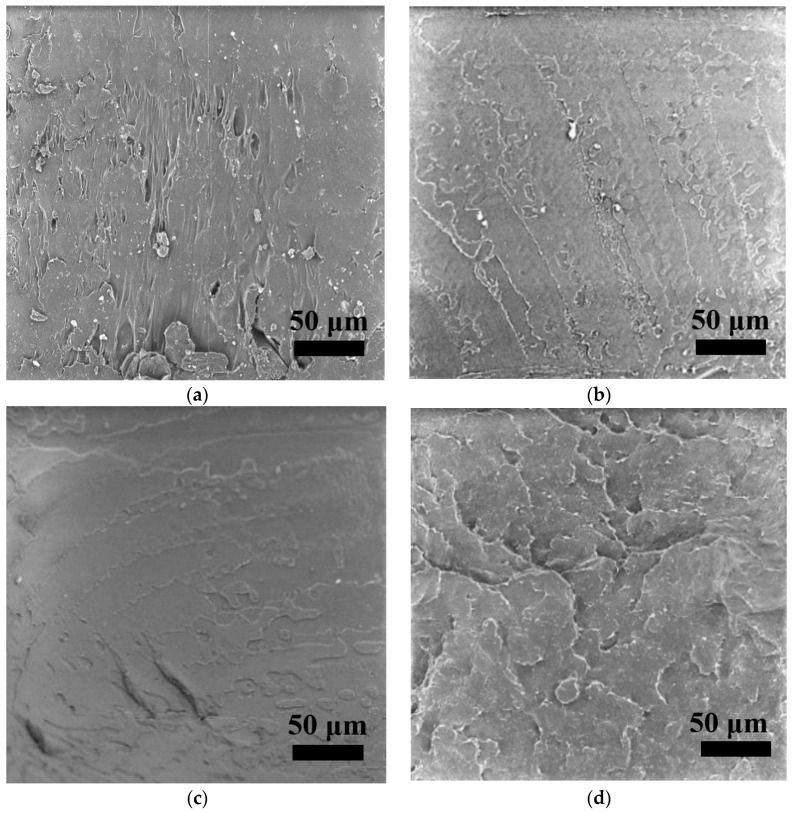

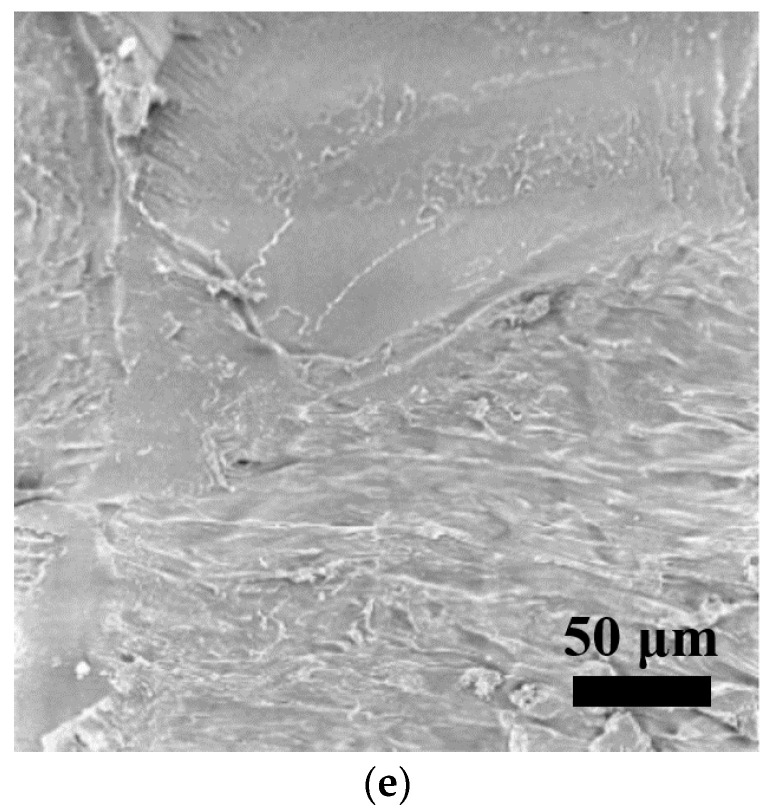
Cross-section morphology of PLA at different soil burial days: (**a**) 0; (**b**) 30; (**c**) 60; (**d**) 90; (**e**) 180.

**Figure 8:**
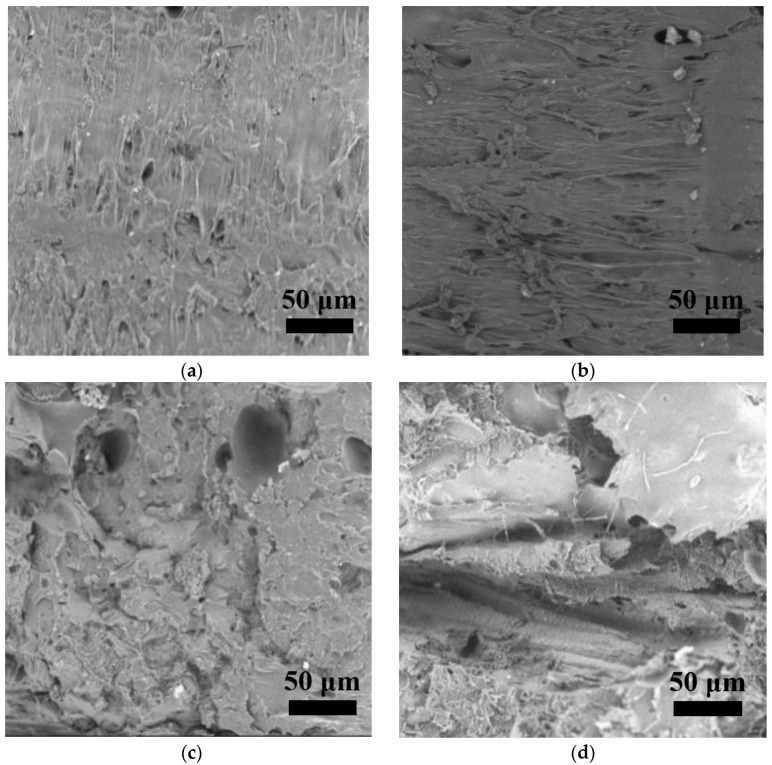

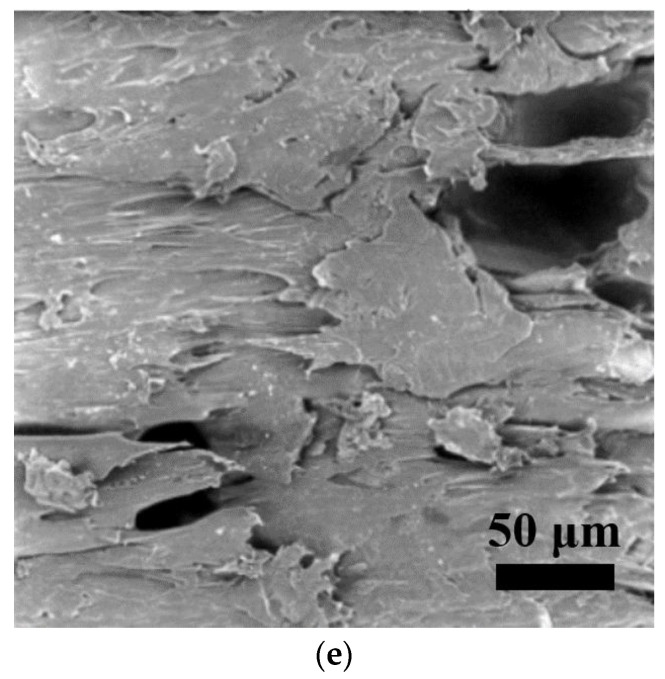
Cross-section morphology of ARP/PLA at different soil burial days: (**a**) 0; (**b**) 30; (**c**) 60; (**d**) 90; (**e**) 180.

Figure 7a–e and Figure 8a–e present the fracture surfaces of FFF 3D-printed PLA and ARP/PLA biocomposite specimens at different soil burial stages, respectively. The PLA before soil burial (Figure 7a) shows a relatively smooth and clear surface, indicating a brittle failure mechanism. After soil burial, the PLA matrix showed a rougher surface of failure, but no microvoids or cracks appeared, indicating that soil burial did not obviously affect the degradation of PLA, which was in agreement with the weight loss and flexural data. Regarding the SEM micrographs of ARP/PLA biocomposites, Figure 8a–e show that the cross-sectional surface became slightly coarse (but not significantly) after soil burial for 30 days (Figure 8b). After being buried in soil for 60 days; however, the fracture surface became much rougher, and some microvoids appeared (Figure 8c). With the continuation of soil burial, much greater voids and cracks could be observed, as shown in Figure 8d,e. These voids and cracks corresponded to the weakened interfacial bonding between the fibers and the polymer matrix. Thus, the SEM micrographs indicate that ARP was not totally miscible with PLA and that the incorporation of ARP accelerated damage to the structure of the printed samples during soil burial.

### 3.5. Thermogravimetric Analysis

**Figure 9:**
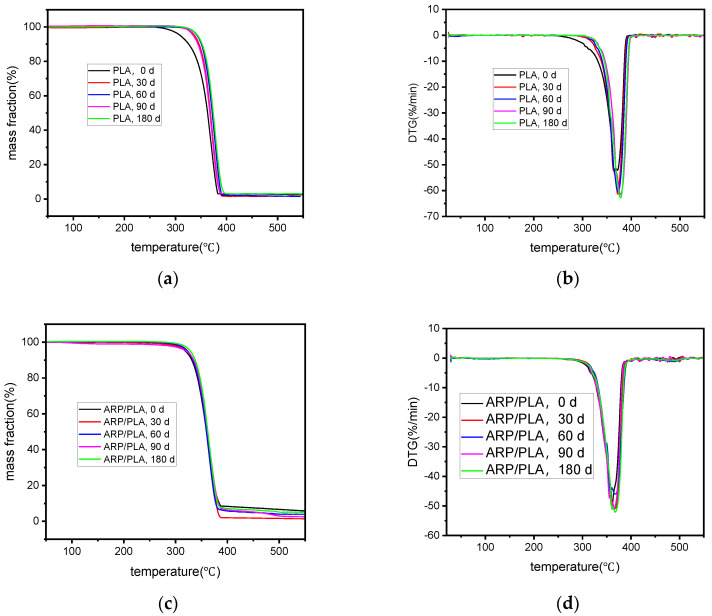
TG–DTG curves of different samples in different degradation periods: (**a**) TG curves of PLA; (**b**) DTG curves of PLA; (**c**) TG curves of ARP/PLA; (**d**) DTG curves of ARP/PLA.

The TGA and DTG thermograms during the decomposition of PLA and ARP/PLA in the temperature range of 20~550 °C are presented in Figure 9a–d. The characteristic thermal degradation parameters are outlined in Table 1. Figure 9 and Table 1 show that not only the incorporation of ARP but also soil burial duration had effects on the thermal stability of the printed samples. The starting thermal degradation temperature (T_i_) of the printed PLA samples before soil burial was 351.1 °C, while the T_i_ value of ARP/PLA decreased to 332.5 °C. The peak temperature at which thermal decomposition took place at the maximum rate (T_p_) ranged from 377.1 to 361.3 °C, meaning that the presence of ARP drastically reduced the thermal stability of PLA. Similar conclusions were previously drawn for some other natural fiber/polymer biocomposites such as cork/PLA [27], durian skin fiber/PLA [28], and rice husk fiber/PLA composites [29].

In terms of the effects of soil burial on the thermal stability of the printed samples, we found that the thermal stability of all samples increased gradually with an extension of the soil burial time. After being buried in soil for 180 days, the T_i_ and T_p_ values of PLA rose to 360.6 and 382.9 °C, respectively, while those of the ARP/PLA biocomposites increased to 339.9 and 368.2 °C, respectively. This trend is analogous to that of rice straw powder/PLA biocomposites [30]. After burial in soil, the components in the 3D-printed samples were easily degraded by water or microorganisms. As a result, the thermal stability increased.

### 3.6. DSC Thermal Analysis

**Figure 10:**
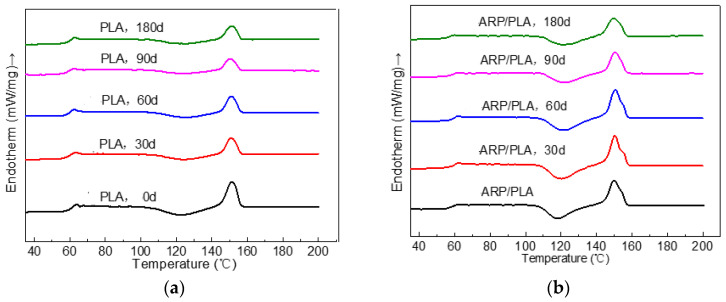
The secondary heating curves of different specimens in different degradation periods. (**a**) PLA; (**b**) ARP/PLA.

Differential scanning calorimetry was used to investigate the glass transition, crystallization, and melting phenomena of PLA and ARP/PLA composites in relation to the effect of soil burial. Figure 10 and Table 2 summarize the DSC behavior and associated thermal parameters for PLA and ARP/PLA. The T_g_ value of PLA was not significantly affected by the addition of ARP. Here, the similar T_g_ value indicates that interactions between ARP and PLA were not strong enough to slow the chain mobility related to the glass transition because of the encompassed geometry and molecular architectures [31]. As shown in Figure 10, pure PLA and ARP/PLA before soil burial were melted with endothermic melting signals at 150.5 and 149.9 °C, respectively, during the second heating scan. Additionally, no obvious differences between the melting points of the two samples could be observed. The T_cc_ of PLA before soil burial was 122.6 °C, but that of ARP/PLA decreased to 118.1 °C. This result suggests the possible effective nucleating role of ARP to accelerate the PLA crystallization process and thus enhance the crystal growth rate. This phenomenon was also observed in wood flour/PBS/PLA composites [25], rice husk/PLA composites [29], and paddy straw/PLA composites [32]. The crystallization percentage of PLA and ARP/PLA was not very high (around 2%), and no definite trend could be found for the effect of soil burial on crystallinity.

## 4. Conclusions

This study investigated the effects of soil burial on the degradation behavior of FFF 3D-printed ARP/PLA. The effects of soil burial time on the material’s physical appearance, weight, morphology, and flexural and thermal properties were examined. For comparison, the effects of soil burial on the physico-mechanical properties of FFF 3D-printed PLA were also investigated. The conclusions are as follows:

(1) The surface color was homogeneous for both PLA and ARP/PLA before soil burial, and no obvious color change was observed on the surface of PLA in the early degradation period. After 60 days, the material’s surface became slightly darkened but not obviously. However, the surface of ARP/PLA became gray with some black spots and crevices throughout the soil burial degradation process, especially after 60 days.
(2) ARP/PLA degraded more rapidly than PLA. We observed a weight reduction of 15.52% for ARP/PLA and weight loss of 1.83% with PLA after keeping the samples for 180 days in soil.
(3) After burying the samples in soil for 180 days, huge drops of 41.01% and 25.49% in the flexural strength and modulus were observed for ARP/PLA composites. The drops were, respectively, 8.64% and 8.06% for plain PLA.
(4) Before soil burial, PLA showed a relatively smooth and clear fracture surface, while ARP/PLA showed a coarse fracture surface and even had some microvoids; after soil burial, the fracture surface of PLA became a little rougher but not obviously. For ARP/PLA, much greater voids and even cracks appeared on the fracture surface.
(5) The thermal stability of all the samples increased gradually with an extension of soil burial time. After being buried in soil for 180 days, the T_i_ and T_p_ values of PLA rose to 360.6 and 382.9 °C, respectively, while those of ARP/PLA biocomposites increased to 339.9 and 368.2 °C, respectively. The effects of soil burial on the thermal properties of ARP/PLA were more serious than the effects on the thermal properties of PLA.

On the basis of the experiment, we confirmed that PLA and ARP/PLA can be subjected to degradation in soil and that the incorporation of ARP can accelerate the degradation of PLA. The results indicated that printed ARP/PLA biocomposites have the potential to be used as sustainable materials with improved biodegradation properties.

## Figures and Tables

**Table 1:** Thermogravimetric analysis information for different specimens.

Sample	Soil Burial Time/Day	Ti/°C	Tp/°C	Char Residue/% (550 °C)
PLA	0	351.1	377.1	2.47
	30	354.3	377.3	2.90
	60	356.2	378.0	1.53
	90	360.0	379.6	2.98
	180	360.6	382.9	1.46
ARP/PLA	0	332.5	361.3	5.76
	30	335.3	365.1	3.95
	60	339.6	366.6	5.03
	90	339.8	367.4	1.39
	180	339.9	368.2	2.52

**Table 2:** DSC thermal information for different specimens.

Sample	Soil Burial Time/Day	Tg/°C	Tcc/°C	Tm/°C	ΔHcc/(J/g)	ΔHm/(J/g)	x_C_ (%)
PLA	0	63.6	122.6	150.5	17.81	19.26	1.74
	30	63.4	123.9	151.0	11.95	13.72	2.12
	60	62.5	124.7	151.2	10.33	11.97	1.97
ARP/PLA	90	62.3	125.2	152.2	7.82	9.19	1.64
	180	62.1	125.9	152.7	8.25	9.65	1.68
	0	62.8	118.1	149.9	22.65	24.44	2.14
	30	62.4	120.3	150.3	23.79	25.36	1.88
	60	62.0	120.8	150.7	23.82	25.61	2.15
	90	61.8	122.0	151.4	17.54	18.84	1.56
	180	60.3	122.1	153.6	15.63	17.93	2.76

## Data Availability

Not applicable.

## Sample Availability

Samples of the compounds are not available from the authors.
